# The C‐type lectin receptor MGL senses *N*‐acetylgalactosamine on the unique *Staphylococcus aureus* ST395 wall teichoic acid

**DOI:** 10.1111/cmi.13072

**Published:** 2019-07-08

**Authors:** Malgorzata E. Mnich, Rob van Dalen, David Gerlach, Astrid Hendriks, Guoqing Xia, Andreas Peschel, Jos A.G. van Strijp, Nina M. van Sorge

**Affiliations:** ^1^ Medical Microbiology University Medical Center Utrecht, Utrecht University Utrecht The Netherlands; ^2^ Glaxo‐Smith Kline Siena Italy; ^3^ Interfaculty Institute of Microbiology and Infection Medicine University of Tübingen Tübingen Germany; ^4^ German Center for Infection Research (DZIF) Tübingen Germany; ^5^ Lydia Becker Institute of Immunology and Inflammation, Division of Infection, Immunity and Respiratory Medicine, Faculty of Biology, Medicine and Health University of Manchester, Manchester Academic Health Science Centre Manchester UK

**Keywords:** C‐type lectin receptor, innate immunity, microbial‐cell interaction, staphylococci, virulence

## Abstract

*Staphylococcus aureus* is a common skin commensal but is also associated with various skin and soft tissue pathologies. Upon invasion, *S*. *aureus* is detected by resident innate immune cells through pattern‐recognition receptors (PRRs), although a comprehensive understanding of the specific molecular interactions is lacking. Recently, we demonstrated that the PRR langerin (CD207) on epidermal Langerhans cells senses the conserved β‐1,4‐linked *N*‐acetylglucosamine (GlcNAc) modification on *S*. *aureus* wall teichoic acid (WTA), thereby increasing skin inflammation. Interestingly, the *S*. *aureus* ST395 lineage as well as certain species of coagulase‐negative staphylococci (CoNS) produce a structurally different WTA molecule, consisting of poly‐glycerolphosphate with α‐O‐*N*‐acetylgalactosamine (GalNAc) residues, which are attached by the glycosyltransferase TagN. Here, we demonstrate that *S*. *aureus* ST395 strains interact with the human Macrophage galactose‐type lectin (MGL; CD301) receptor, which is expressed by dendritic cells and macrophages in the dermis. MGL bound *S*. *aureus* ST395 in a *tagN*‐ and GalNAc‐dependent manner but did not interact with different *tagN*‐positive CoNS species. However, heterologous expression of *Staphylococcus lugdunensis tagN* in *S*. *aureus* conferred phage infection and MGL binding, confirming the role of this CoNS enzyme as GalNAc‐transferase. Functionally, the detection of GalNAc on *S*. *aureus* ST395 WTA by human monocyte‐derived dendritic cells significantly enhanced cytokine production. Together, our findings highlight differential recognition of *S*. *aureus* glycoprofiles by specific human innate receptors, which may affect downstream adaptive immune responses and pathogen clearance.

## 1 INTRODUCTION

*Staphylococcus aureus* is a common member of the human microbiome and colonises up to 30% of the population, where it mostly resides in the nares and on the skin (Eriksen, Espersen, Rosdahl, & Jensen, 1995; Kluytmans, van Belkum, & Verbrugh, 1997; Wertheim et al., 2005). *S*. *aureus* is a leading cause of surgical site infections and skin infections as well as health care‐associated pneumonias (Pozzi et al., 2017). Treatment of infections is hampered by the continuous emergence of antimicrobial resistance, most prominently methicillin‐resistant *S*. *aureus* and vancomycin‐resistant *S*. *aureus* (Weigel et al., 2003, Lakhundi & Zhang, 2018). Understanding the molecular mechanisms underlying different *S*. *aureus* infections will support the development of new treatment strategies including vaccines.

Components of the bacterial cell envelope are critical for *S*. *aureus* host‐pathogen interaction, both at the level of colonisation but also during systemic infection by evading host immune responses (Weidenmaier & Lee, 2016). One of the most abundant and exposed structures on the Gram‐positive cell wall is wall teichoic acid (WTA). WTA is a glycopolymer that is covalently bound to peptidoglycan. WTA is critical for *S*. *aureus* physiology and infection biology through its role in cation sequestration, horizontal gene transfer by bacteriophages, and adherence function to human nasal epithelial cells (Weidenmaier & Peschel, 2008, Swoboda, Campbell, Meredith, & Walker, 2010, Winstel et al., 2013). In the majority of *S*. *aureus* strains, WTA is composed of a poly‐ribitolphosphate (RboP) backbone decorated with positively charged D‐alanine and *N*‐acetyl‐D‐glucosamine (GlcNAc) residues. Synthesis of the WTA RboP backbone and its modification is orchestrated by *tar* genes. *tarM*, *tarS*, and *tarP* encode specific glycosyltransferases that catalyse the attachment of GlcNAc residues. TarM adds α‐GlcNAc residues at C4 hydroxyl groups of RboP, whereas TarS and TarP modify RboP with β‐GlcNAc residues at C4 or C3 hydroxyl groups, respectively (Brown et al., 2012; Gerlach et al., 2018; Xia et al., 2010). The WTA α‐ and β‐GlcNAc modifications impact interactions of *S*. *aureus* with both innate and adaptive immune components, including mannose‐binding lectin, langerin, and antibodies (Park et al., 2010; Kurokawa et al., 2013; Lee et al., 2015; Gerlach et al., 2018; van Dalen et al., 2019).

Not all *S*. *aureus* strains express structurally identical WTA. In contrast to the common RboP‐GlcNAc WTA, *S*. *aureus* isolates of the ST395 lineage produce WTA composed of a poly‐glycerolphosphate (GroP) backbone decorated with α‐*N*‐acetyl‐D‐galactosamine (α‐GalNAc) residues, which are attached by glycosyltransferase TagN (Winstel et al., 2013; Winstel, Sanchez‐Carballo, Holst, Xia, & Peschel, 2014). The synthesis of this structurally different WTA impacts recognition and horizontal gene transfer by phages (Winstel et al., 2013; Winstel et al., 2014). Interestingly, GroP‐GalNAc WTA is also produced by several coagulase‐negative staphylococci (CoNS), which are also common inhabitants of skin but are generally less associate with skin pathologies compared with *S*. *aureus* (Endl, Seidl, Fiedler, & Schleifer, 1983; Winstel et al., 2013; Winstel et al., 2014).

C‐type lectin receptors (CLRs) are a family of pattern‐recognition receptors that are dedicated to sense both self and non‐self glycan structures through their characteristic carbohydrate recognition domains (CRDs; Brown, Willment, & Whitehead, 2018). CLRs have a particular expression pattern on subsets of immune cells. We recently identified that the CLR langerin (CD207), which is exclusively expressed on Langerhans cells in the skin epidermis, interacts with *S*. *aureus* through WTA β‐1,4‐GlcNAc, which affects Langerhans cell responses and skin inflammation in mice (van Dalen et al., 2019). In contrast, *S*. *aureus* ST395 does not interact with langerin (van Dalen et al., 2019). However, both dermal dendritic cells (DCs) and dermal macrophages express the trimeric CLR macrophage galactose‐type lectin (MGL; CD301), which recognises terminal GalNAc residues as a result of a Gln‐Pro‐Asp motif in its CRD (Tanaka et al., 2017). GalNAc is incorporated into, among others, pathogen‐produced lipo‐oligosaccharides from *Campylobacter jejuni* and *Neisseria gonorrhoeae* (van Sorge et al., 2009; van Vliet et al., 2009), and confers binding to MGL in a Ca^2+^‐dependent manner, inducing uptake and cellular responses (van Liempt et al., 2007). We therefore hypothesised that *S*. *aureus* ST395 might also be recognised by MGL via α‐GalNAc modifications on WTA and may impact downstream immune responses.

Using recombinant MGL constructs, we demonstrate that human MGL and mouse MGL2 interact with *S*. *aureus* ST395 WTA in a α‐GalNAc‐ and *tagN*‐dependent manner. Interestingly, *tagN*‐encoding CoNS did not interact with MGL, although heterologous expression in a *tagN*‐deficient *S*. *aureus* background proves their function as GalNAc transferases. Importantly, loss of *tagN* in *S*. *aureus* ST395 attenuates production of specific cytokines by human monocyte‐derived dendritic cells (moDCs).

## 2 RESULTS

### 2.1 Human MGL interacts with S. aureus ST395 strains in a tagN‐dependent manner

**Figure 1:**
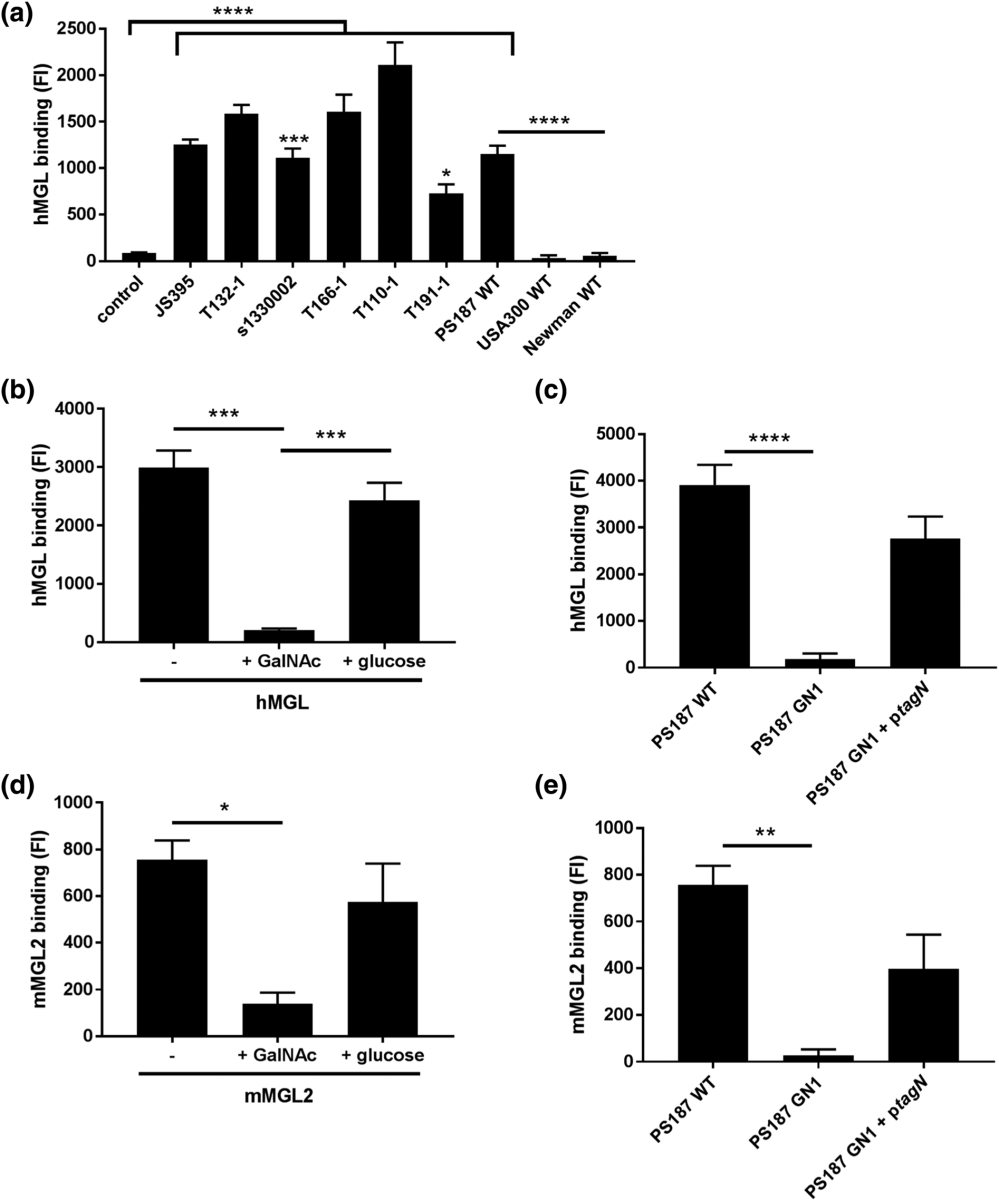
Human and mouse macrophage galactose‐type lectin (MGL) interact with Staphylococcus aureus ST395 strains in a tagN‐dependent manner. (a) hMGL binding to different S. aureus ST395 lineage strains, USA300 wild‐type (WT) and Newman WT detected by anti‐hisTag‐FITC antibody. Control represents S. aureus PS187 WT incubated with secondary detection antibody. (b and d) Interaction between (b) hMGL or (d) mMGL2 to S. aureus PS187 WT in the absence or presence of GalNAc (50 mM) or glucose (50 mM). (c and e) Binding of (c) hMGL or (e) mMGL2 to PS187 WT, GN1, GN1 + ptagN and two non‐ST395 strains. Means of geometric mean fluorescence intensity ± standard error of mean from three independent experiments are shown. *p < .05, **p < .01, ***p < .005, ****p < .0001

Human MGL is the only CLR family member with specificity for α‐GalNAc (van Vliet et al., 2005). Because the *S*. *aureus* ST395 lineage produces GalNAc‐decorated WTA, we investigated whether *S*. *aureus* ST395 was recognised by human MGL. Using flow cytometry, we tested multiple *S*. *aureus* isolates from the ST395 lineage for binding to recombinant soluble his‐tagged MGL. All strains of this lineage bound MGL, whereas no interaction was observed with USA300 and Newman strains (non‐ST395 strains), which both express RboP‐GlcNAc WTA (Figure 1a). Interestingly, the levels of MGL binding varied for different ST395 strains (Figure 1a), likely reflecting different expression levels of the MGL ligand. Addition of soluble GalNAc prevented interaction of MGL with ST395 strain PS187, whereas similar levels of glucose did not affect binding (Figure 1b), indicating that binding occurs through the MGL CRD. To confirm that the interaction between *S*. *aureus* ST395 and MGL depends on WTA GalNAc, we assessed binding of MGL to wild‐type (WT) PS187 and the isogenic mutant strain GN1, which lacks the C‐terminal glycosyltransferase domain of *tagN* and is consequently deficient for WTA α‐GalNAc (Winstel et al., 2014). MGL binding was lost in the *tagN*‐deficient mutant and could be restored by complementation with plasmid‐expressed full‐length *tagN* (Figure 1c), confirming that WTA α‐GalNAc of *S*. *aureus* ST395 is the ligand of MGL.

We have previously observed that langerin shows a certain level of species specificity, that is, mouse langerin does not interact with *S. aureus* (van Dalen et al., 2019). Therefore, we investigated interaction of PS187 with mouse homologue MGL2 (Singh et al., 2009). Like human MGL, mouse MGL2 interacted with PS187, could be blocked with GalNAc, and interaction was lost upon deletion of *tagN* (Figure 1d,e), suggesting that the interaction is, at least partially, conserved across species.

### 2.2 S. lugdunensis tagN encodes a GalNAc‐transferase that produces a MGL ligand

**Figure 2:**
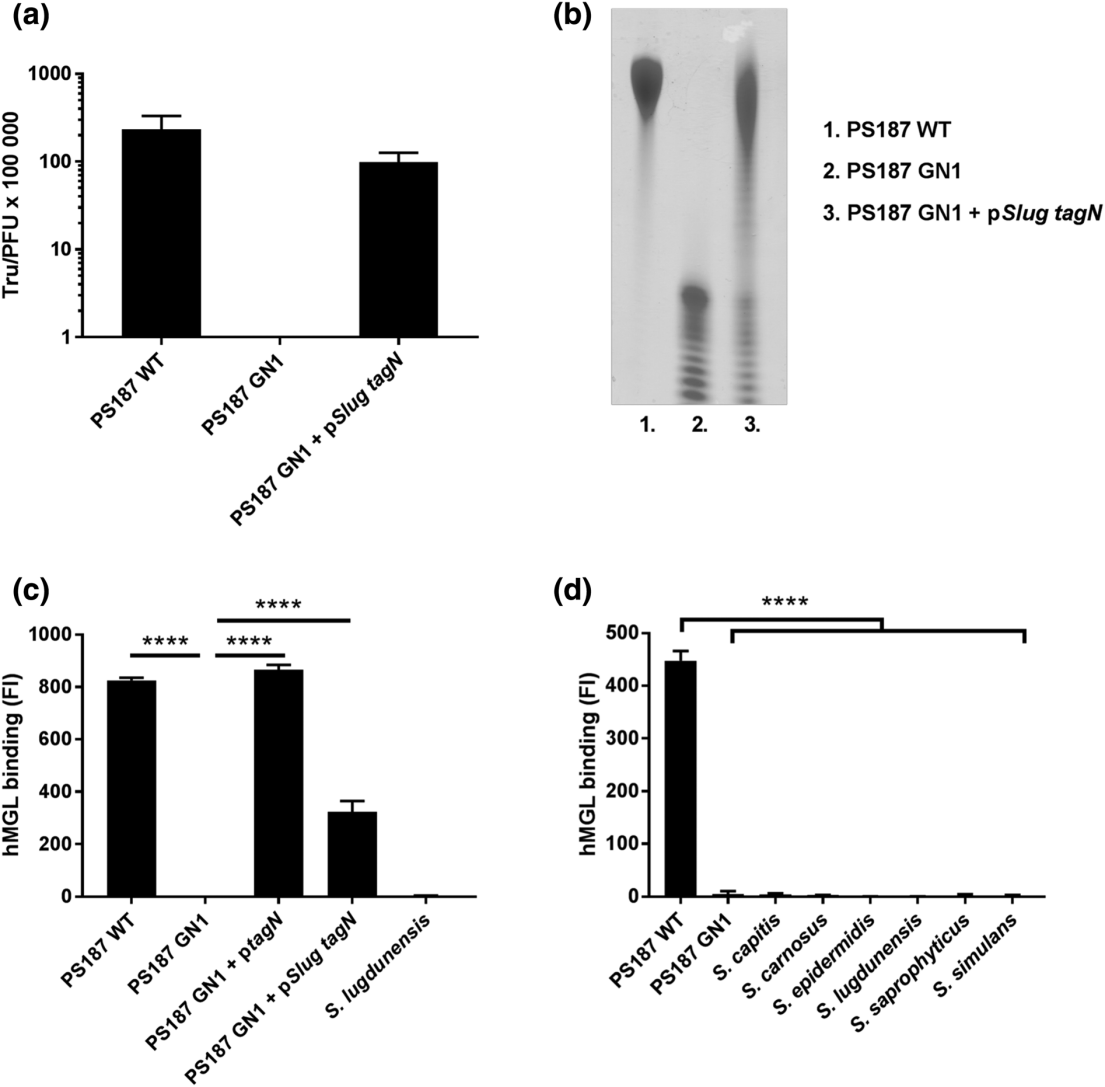
Staphylococcus lugdunensis tagN encodes a GalNAc‐transferase that produces a macrophage galactose‐type lectin (MGL) ligand. (a) Transfer of SaPI BovI via phage **ϕ**187 into PS187 wild‐type (WT), GN1 mutant, and GN1 complemented with tagN from S. lugdunensis (pSlug tagN). Values are displayed as transductants per plaque‐forming units (TrU/PFU). In case of GN1 no transductants were obtained. (b) PAGE analysis of wall teichoic acid from Staphylococcus aureus PS187 WT, GN1 mutant, and GN1 complemented with tagN from S. lugdunensis (pSlug tagN). (c) Binding of hMGL to S. aureus PS187 WT, GN1 mutant, and GN1 complemented with either PS187 tagN (ptagN) or pSlug tagN. (d) Interaction of different coagulase‐negative staphylococci species with hMGL. Means of geometric mean fluorescence intensity ± standard error of mean from three independent experiments are shown. ****p < .0001

Similar to *S*. *aureus* ST395 WTA, certain CoNS species express GroP‐type WTA. In addition, several CoNS species express homologues of the *tagN* gene, suggesting that CoNS may decorate WTA in a similar fashion as *S*. *aureus* ST395 strains (Winstel et al., 2014). Indeed, complementation of PS187 GN1 with a *tagN* homologue from *Staphylococcus carnosus* restores GalNAc glycosylation and phage susceptibility (Winstel et al., 2014). Similarly, we were able to confer susceptibility to phage ϕ187 by complementing the GN1 mutant, for which no transductants were obtained, with *tagN* from *Staphylococcus lugdunensis* (Figure 2a). In addition, the WTA migration of this complemented strain was similar to that of WTA from PS187 WT (Figure 2b). Importantly, heterologous expression of *S*. *lugdunensis tagN* in PS187 GN1 also restored binding to MGL (Figure 2c). In contrast, none of the CoNS species that contain a *tagN* homologue interacted with MGL (Figure 2d), despite reactivity with the GalNAc‐specific plant lectin SBA for *Staphylococcus carnosus*, *Staphylococcus capitis*, and *Staphylococcus saprophyticus* (Figure S1). These data suggest that *S*. *lugdunensis tagN* encodes a GalNAc transferase. However, it is likely not or only lowly expressed in *S*. *lugdunensis* in our culture conditions.

### 2.3 S. aureus PS187 interacts with and activates human moDCs

**Figure 3:**
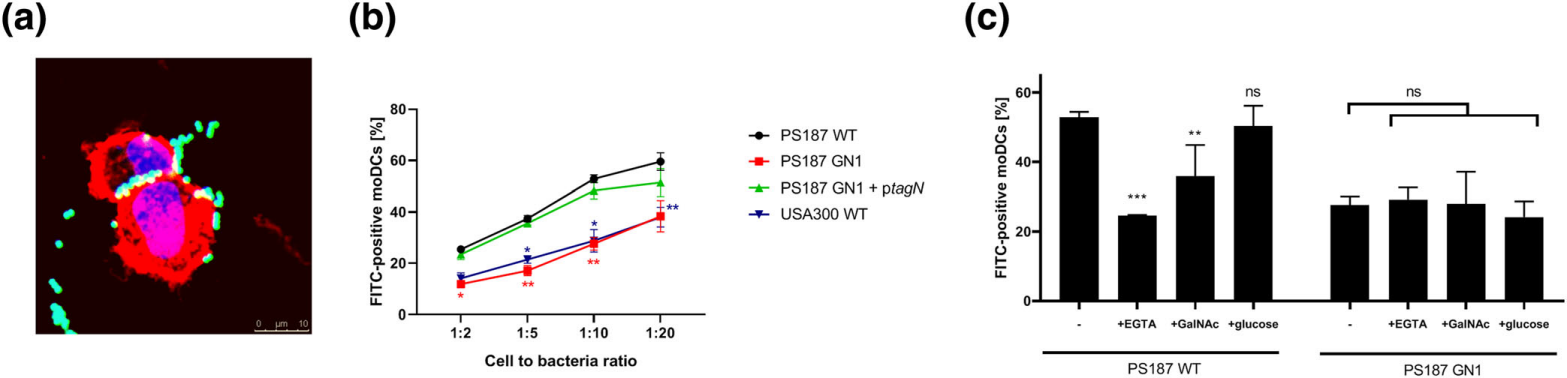
Wall teichoic acid‐GalNAc contributes to interaction between human monocyte‐derived dendritic cells (moDCs) and Staphylococcus aureus PS187. (a) Binding of FITC‐labeled S. aureus PS187 WT (green) to immature moDCs (membrane in red, nucleus in blue). Cytospin samples were prepared from cell suspensions incubated with bacteria in 1:50 ratio for 30 min. (b) Binding of FITC‐labeled S. aureus strains to moDCs at different cell‐to‐bacteria ratios after 30 min of incubation. Data are presented as mean ± standard error of mean (SEM) of percentage of FITC‐positive moDCs (n = 3). Significance shown as compared with binding of PS187 WT to moDCs within certain ratio in red for dGN1 mutant and in blue for UAS300 WT. (c) Binding of FITC‐labeled S. aureus PS187 WT and GN1 to moDCs in 1:10 cell‐to‐bacteria ratio after 30 min in the absence or presence of 1 mM EGTA, 50 mM GalNAc or 50 mM glucose (control). Data are presented as a mean ± SEM of percentage of FITC‐positive moDCs (n = 3). *p < .05, **p < .01, ***p < .005

MGL is expressed on a range of immune cells including human DCs and macrophages residing in skin and lymph nodes, blood CD1c + DCs, and immature moDCs (van Vliet, Gringhuis, Geijtenbeek, & van Kooyk, 2006, Schutz & Hackstein, 2014, Heger et al., 2018). To investigate the interaction of MGL with *S*. *aureus* ST395 strains in a more biologically relevant system, we used a cell‐based assay with human immature moDCs. Fluorescein isothiocyanate (FITC)‐labeled *S*. *aureus* PS187 WT bound readily and in a ratio‐dependent manner to moDCs (Figure 3a,b). Interestingly, binding was reduced for the *tagN*‐deficient mutant and USA300 strains, which both do not express GalNAc on their surface (Figure 3b). Binding to moDCs was restored to WT levels in the *tagN*‐complemented strain (Figure 3b). Complementary, we assessed the effect of different blocking agents, that is, ethylene glycol‐bis(β‐aminoethyl ether)‐N,N,N',N'‐tetraacetic acid (EGTA), GalNAc, and glucose (as a control; Figure 3c). Binding of PS187 WT, but not of the GN1 mutant, was reduced upon coincubation of EGTA and GalNAc, but not glucose (Figure 3c). These data demonstrate that the PS187‐moDC interaction is partially preventable by addition of GalNAc or calcium scavenging, which is in line with a possible role for MGL.

**Figure 4:**
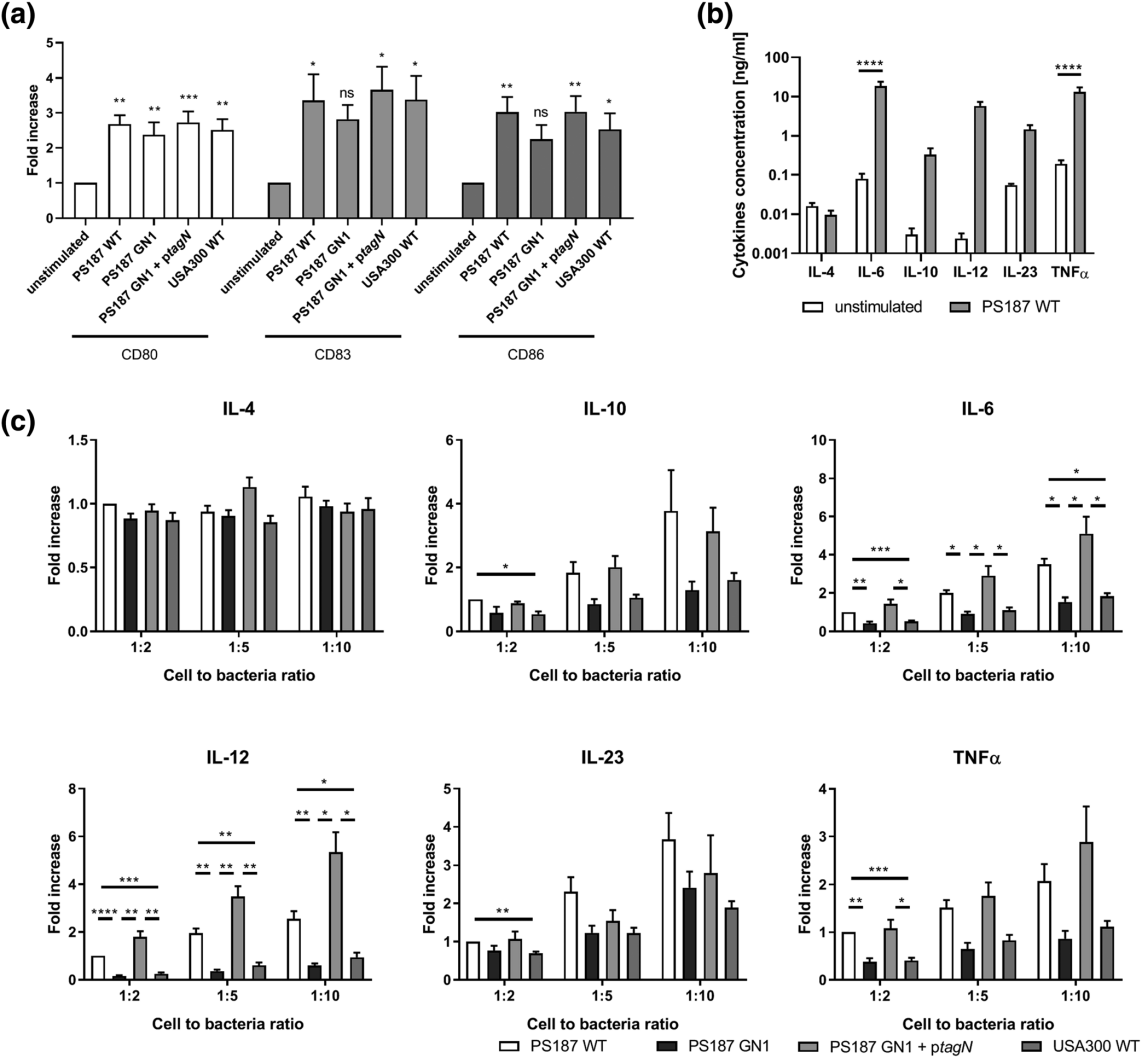
Human monocyte‐derived dendritic cells (moDCs) are activated by Staphylococcus aureus PS187 and cytokine production is affected by wall teichoic acid‐GalNAc. (A) Relative expression of surface maturation markers on moDCs after stimulation with gamma‐irradiated S. aureus strains at cell‐to‐bacteria ratio of 1:10 for 16 hr. Data are presented as fold change ± standard error of mean (SEM) relative to unstimulated control (n = 3 donors). (B) Cytokine expression by moDCs after 16 hr of incubation without or with gamma‐irradiated S. aureus PS187 WT in 1:10 cell‐to‐bacteria ratio. Data are presented as mean of cytokine concentration ± SEM (n = 6 donors). (C) Cytokine expression by moDC after incubation with gamma‐irradiated S. aureus strains in 1:2, 1:5, and 1:10 cell‐to‐bacteria ratio for 16 hr. Data are presented as mean of fold increase over PS187 WT 1:2 ± SEM (n = 6 donors). *p < .05, **p < .01, ***p < .005, ****p < .0001

Loss of interaction with MGL may affect immune activation of moDCs, such as expression of costimulatory molecules or cytokine production, resulting in different immunological responses. We therefore investigated moDCs maturation and cytokine production after stimulation with gamma‐irradiated *S*. *aureus* PS187 WT, GN1, *tagN*‐complemented GN1, or USA300 WT for 16 hr. MoDCs upregulated maturation markers CD80, CD83, CD86, and CD40, indicating that all *S*. *aureus* strains activate moDCs (Figure 4a, Figure S2). We observed little effect on expression of HLA‐DR except with PS187 WT (Figure S2). However, there was no difference in the induction of moDC maturation by the different *S*. *aureus* strains (Figure 4a, Figure S2). We also analysed moDC cytokine production. *S*. *aureus* PS187 WT induced expression of IL‐6, IL‐12p70, IL23p19, IL‐10, and TNFα, but not IL‐4 when incubated with moDCs (Figure 4b). Interestingly, at a cell‐to‐bacteria ratio of 1:2, cytokine production was significantly lower when strains did not produce GalNAcylated WTA, that is, PS187 GN1 and USA300 WT (Figure 4c). At higher ratios, this difference was robust for IL‐6 and IL12p70 and trends remained for IL‐10, IL23p19, and TNFα (Figure 4c). Cytokine production by moDCs was restored to PS187 WT levels in cells stimulated with the *tagN*‐complemented strain (Figure 4c). Overall, these data indicate that the production of select pro‐inflammatory cytokines, that is, IL‐6 and IL12p70, by moDCs is enhanced by recognition of the α‐GalNAc modifications present on *S*. *aureus* PS187 WTA.

To determine whether differences in cytokine production are not just WTA GalNAc‐dependent but also MGL‐dependent, we attempted to block the interaction using a commercially available anti‐MGL blocking antibody. These experiments are technically complicated by the presence of protein A and Sbi on the *S*. *aureus* surface, as these proteins bind IgG Fc, thereby possibly increasing DC interaction by binding to the blocking antibody. MoDC cytokine production in response to PS187 WT was not affected by the presence of either the blocking antibody or the isotype control antibody compared with bacteria alone (Figure S3). We confirmed that incubation of the antibodies with moDCs by itself did also not significantly affect cytokine production (Figure S4). Therefore, we are currently unable to prove that moDC cytokine production in response to *S*. *aureus* PS187 occurs through MGL.

## 3 DISCUSSION

Here, we show the molecular interaction between WTA of *S*. *aureus* ST395 and MGL, an innate receptor of the CLR family. This interaction is dependent on α‐GalNAc modifications of *S*. *aureus* WTA and contributes to increased cytokine production in MGL‐expressing moDCs. Although Winstel et al. showed the importance of *S*. *aureus* GalNAc glycosylation for phage infection (Winstel et al., 2014), there was no previous indication for interaction with human receptors. Because the ST395 lineage is present in nasal and blood culture isolates (Holtfreter et al., 2007), interaction with MGL may be biologically relevant in context of recognition and clearance by the immune system.

This is the first identification of MGL interaction with a Gram‐positive bacterium. Previous studies have identified MGL ligands on the surface of Gram‐negative pathogens, including *C*. *jejuni*, *N*. *gonorrhoeae*, and *Escherichia coli* strain R1 (Maalej et al., 2019; van Sorge et al., 2009; van Vliet et al., 2009). For *Escherichia coli* strain R1, soluble lipo‐oligosaccharide (LOS) was identified as a ligand for recombinant human MGL, although no functional consequences were assessed (Maalej et al., 2019). For *C*. *jejuni*, MGL ligands are GalNAc residues incorporated in LOS and *N*‐glycosylated proteins (van Sorge et al., 2009). For *N*. *gonorrhoeae*, the ligand is a terminal GalNAc residue on the LOS of phenotype C strains, which influenced binding to moDCs and subsequent T helper differentiation (van Vliet et al., 2009). Similarly, our data show that loss of GalNAc on the *S*. *aureus* surface decreases binding to moDCs, which corresponds with assays using recombinant MGL. Importantly, binding of GN1 to moDCs could be restored by *tagN* complementation, suggesting that one of the involved receptors is MGL. Experiments using the calcium chelator EGTA and GalNAc monosaccharide also decreased moDC binding, although GalNAc had only a modest inhibiting effect. This may implicate the involvement of other calcium‐dependent, GalNAc‐independent receptors in the interaction between *S*. *aureus* PS187 and DCs.

Presence of the GalNAc‐WTA epitope also affected DC cytokine production, especially increasing production of IL‐6 and IL‐12p70 across the tested range of bacteria‐to‐cell ratios. This is in contrast to observations with *C*. *jejuni*, where absence of the MGL ligand on glycosylated proteins actually increased DC cytokine production, suggesting that MGL triggering dampened immune activation. These contrasting outcomes suggest that the context in which MGL is engaged influences how MGL affects DC responses. Indeed, previous reports have indicated that MGL triggering alone does not induce high cytokine secretion by CD1c + DCs but requires costimulation with Toll‐like receptor ligands to differentially affect IL‐8, IL‐10, and TNFα production (Heger et al., 2018; van Vliet et al., 2013). Because Toll‐like receptor ligands differ between Gram‐positive and Gram‐negative bacteria, this may explain the different effects on DC cytokine production that we observe here. Alternatively, we can speculate that observed differences in DC cytokine production are not completely MGL‐dependent. It cannot be excluded that additional receptors were triggered in the absence of WTA‐GalNAc as a result of newly exposed structures on the *S*. *aureus* surface. Additionally, other receptors may be more important for induction of cytokines, which is also implied by the experiments using anti‐MGL blocking antibodies, which did not affect cytokine production.

It has been well established that MGL binds to galactose‐ and GalNAc‐rich terminal motifs in a Ca^2+^‐dependent manner (Suzuki, Yamamoto, Toyoshima, Osawa, & Irimura, 1996; van Vliet et al., 2005). These modifications are often found in the extracellular matrix of host tissues. Interaction of DCs with extracellular matrix via MGL inhibits their migration from dermis to the lymph nodes. Therefore, the presence of MGL‐expressing DCs in the skin might be explained by the abundance of GalNAc epitopes in dermal tissues (van Vliet, Paessens, Broks‐van den Berg, Geijtenbeek, & van Kooyk, 2008). Recognition of GalNAc‐decorated bacteria, such as *S*. *aureus* PS187, by dermal DCs can disrupt the interaction with the extracellular matrix, which consequently would allow DCs to migrate to the lymph nodes to present antigen. In addition, the observation that the detection of GalNAc‐epitopes enhances cytokine production additionally suggests that this interaction is an immune defense strategy and likely not a part of the immune evasion repertoire of *S*. *aureus*.

When dermal DCs encounter pathogens and migrate to the lymph nodes to present antigens, the final step is to develop an adaptive immune response to eradicate these pathogens. Previous studies have demonstrated high levels of IgG antibodies against WTA GlcNAc modifications in human serum, indicating the importance of this epitope in adaptive immunity. Specifically, the anti‐WTA β‐1,4‐GlcNAc antibodies induce complement activation and opsonophagocytosis of *S*. *aureus* strains expressing a RboP‐GlcNAc WTA (Lee et al., 2015). Because *S*. *aureus* ST395 expresses an alternative WTA structure with a different glycosylation pattern, it will be of interest to study whether antibody responses are mounted against this specific WTA glycoepitope and whether these antibodies facilitate *S*. *aureus* phagocytosis and killing.

Surprisingly, our data did not show any binding of MGL to CoNS. This could simply reflect lack of *tagN* gene expression under the tested growth conditions. Alternatively, it may indicate that additional factors such as epitope density, capsule expression or overall accessibility prevent MGL interaction, which are potentially different in *S*. *lugdunensis* compared with *S*. *aureus*. The observation that heterologous expression under a constitutive promoter confers both susceptibility to phage ϕ187 as well as MGL binding does not exclude either possibilities, but does confirm that *tagN* from *S*. *lugdunensis* encodes an α‐GalNAc transferase. The observed discrepancy between SBA and MGL binding to several CoNS suggests that, despite high homology between *S*. *aureus* and CoNS *tagN* genes, the pattern or specificity of WTA GalNAc decoration may be slightly different, thereby preventing interaction for MGL.

In summary, we have demonstrated that *S*. *aureus* ST395 lineages engage the receptor MGL and induce maturation and cytokine production of human DCs, which is partially dependent on expression of WTA‐GalNAc. Together with the previous findings that RboP‐GlcNAc interacts with langerin, these findings create an overall view that the *S*. *aureus* WTA glycosylation profile dictates the interaction with specific innate immune receptors on antigen‐presenting cells, which may have important consequences for immune defense and pathogen clearance.

## 4 EXPERIMENTAL PROCEDURES

### 4.1 Bacterial strains

Bacteria (Table S1) were grown either on Todd Hewitt (Oxoid) agar or in Todd Hewitt broth supplemented with chloramphenicol (Sigma‐Aldrich) at a concentration 10 μg/ml when required. For all experiments, bacteria were grown overnight, subcultured the next day in fresh Todd Hewitt broth, and grown to exponential phase (optical density at 600 nm [OD_600_] = 0.6) for use in experiments.

### 4.2 Molecular cloning

*TagN* was amplified using primer pair N474‐slug‐bam (up; 5′‐ATCGGATCCAAAGGAGGTATTATAATGGCATTAAAGAAATTTATAATTAATCA‐3′) and N474‐slug‐Eco(dn;5′‐GAGAGAATTCCTATTTAAGTAGCTTATAAAATTCATTA‐3′) and genomic DNA of *S*. *lugdunensis* HKU09‐01 as template. The amplicon was cloned into shuttle‐vector pRB474 (Bruckner, 1992) via the BamHI and EcoRI restriction sides.

### 4.3 SaPI transfer assay

Lysate of SaPIbov1 (ϕ187) bearing a tetracyclin resistance marker was generated as previously described (Winstel et al., 2014). In brief, overnight culture of PS187 SaPIbov1::tet (final OD 0.1) of was incubated with ϕ187 (final concentration of 10^7^ plaque forming units (PFU)/mL) in a final volume of 10 mL TSB for 30 min at 37°C and subsequently at 30°C until visible bacterial lysis. The obtained lysate was centrifuged and filtered (pore size 0.2‐0.45 μm). SaPI transfer was performed by mixing 100 μL of SaPI lysate with 200 μL of stationary bacteria (OD = 0.5) and subsequent incubation for 15 min at 37°C. The mixture was centrifuged for 3 min at 10,000 g and plated on Tryptic soy agar (TSA) plates supplemented with 3 μg/mL tetracycline. Plates were incubated overnight at 37°C, and transductants were enumerated.

### 4.4 WTA isolation and analysis by polyacrylamide gel electrophoresis (PAGE)

WTA was isolated as previously described (Winstel et al., 2013). Briefly, overnight culture of *S*. *aureus* PS187 was grown in BM (0.5 % w/v yeast extract; 1% w/v Soy peptone; 0.5% NaCl; 0.1 % K2HPO3) supplemented with 0.25% w/v glucose was harvested by centrifugation and washed using ammonium acetate buffer (AAB, 20 mM, pH 4.8). Bacterial cells were opened using a Euler cell mill (2.5 mL AAB/4.5 glass beads/1 g cell pellet). The obtained lysate was digested overnight with RNAse and DNAse at 37°C, subsequently treated by ultrasonification, and incubated with 2% sodium dodecyl sulfate (SDS) for 1 hr at 60°C. Purified peptidoglycan was washed extensively with AAB. WTA was released by 5% tri chloroacetic acid (TCA) treatment for 4 hr at 60°C. The supernatant was neutralised using NaOH and dialyzed against ddH2O.

PAGE analysis of WTA occurred as previously described (Xia et al., 2010). WTA samples (400 nmol phosphate) were applied to a polyacrylamide gel (26%) and separated electrophoretically for 13 hr at 25 mA. WTA bands were visualised using Alcian blue (0.005%) in staining solution (40% ethanol, 5% acetic acid).

### 4.5 Lectin binding assay

Bacteria were harvested by centrifugation (4,000×g, 10 min) and resuspended to OD_600_ of 0.4 in Tris buffer (20 mM Tris [Roche], 150 mM NaCl [Sigma‐Aldrich], 2 mM CaCl_2_·2 H_2_O [Merck], 2 mM MgCl_2_·6 H_2_O [Merck], pH 7.0; TSM) with 0.1% bovine serum albumin (BSA, Merck). Bacteria were incubated with 5 μg/ml of recombinant human MGL‐his (R&D Systems), 10 μg/ml of recombinant mouse MGL2‐his (R&D Systems), 4 μg/ml of SBA‐FITC (soy bean agglutinin, Vector Laboratories), or 2 μg/ml of sWGA‐FITC (succinylated wheat germ agglutinin, Vector Laboratories). Binding of recombinant human MGL and murine MGL2 was detected using anti‐hisTag FITC‐conjugated antibodies (LifeSpan BioSciences). For blocking, we used soluble *N*‐acetyl‐D‐galactosamine (Fluka, Sigma‐Aldrich) or glucose (Merck) at 50 mM. Samples were analysed using flow cytometry (FacsVerse, BD Biosciences).

### 4.6 Isolation of human monocytes and differentiation to immature DCs

Buffy coats from healthy anonymous donors were purchased from Sanquin Amsterdam and obtained according to the good clinical practice in accordance with the declaration of Helsinki. Donors have given their written consent to the study. Peripheral blood mononuclear cells (PBMCs) were isolated from buffy coats using Ficoll‐Paque PLUS (GE Healthcare) density gradient and monocytes were obtained as described in Sallusto and Lanzavecchia (1994). Briefly, harvested PBMCs were washed twice with RPMI 1640 (Lonza) supplemented with 5% foetal bovine serum (FBS, Biowest). Monocytes were further isolated from the PBMC fraction using density gradient of 60%, 47.5 %, and 34 % Percoll (Sigma‐Aldrich) in RPMI 1640 + 10% FBS. Harvested monocytes were washed three times with RPMI 1640 + 5% FBS and incubated at the concentration 0.5 × 10^6^ cells/ml with differentiation medium consisting of RPMI 1640 supplemented with 10% HyClone FBS (GE Healthcare), 800 IU/ml GM‐CSF (Bio Connect), 250 IU/ml IL‐4 (Thermo Fisher Scientific), 100 IU/ml penicillin‐streptomycin, and 2.4 mM L‐glutamine for 5 to 7 days to obtain immature DCs.

### 4.7 Binding of FITC‐labeled bacteria to moDCs

To perform bacteria binding assays, *S*. *aureus* strains were labelled with FITC (Sigma‐Aldrich). Five milligrams of bacterial culture in exponential phase were pelleted and resuspended in cold PBS with 0.1% BSA. Bacteria were incubated with 0.5 mg/ml FITC for 30 min on ice protected from light. Bacteria were washed three times with cold PBS + 0.1% BSA supplemented with 1% ammonia and resuspended in TSM + 0.1% BSA at OD_600_ of 0.4.

Immature moDCs were harvested by centrifugation and re‐suspended in TSM + 0.1% BSA (1 × 10^6^ cells/ml). Cells were incubated with bacteria at 1:2, 1:5, 1:10, and 1:20 cell to‐bacteria ratios in a 96‐well round bottom plate for 30 min in 4°C protected from light. For blocking, cells were preincubated for 15 min at room temperature with 1 mM EGTA (Brunschwig Chemie), 50 mM GalNAc (Fluka, Sigma‐Aldrich), or 50 mM glucose (Merck). Next, cells were incubated with bacteria at 1:10 cell‐to‐bacteria ratio for 30 min at 4°C, protected from light. Samples were washed with TSM + 1% BSA, fixed using 1% formaldehyde in PBS, and analysed using flow cytometry. Microscopy pictures were prepared using 1:50 cell to bacteria ratio suspensions. Cells were attached to the glass slides using a Shandon Cytospin 3 centrifuge. Cellular membranes were stained using WGA‐Alexa Fluor 647 (Thermo Fisher Scientific), cell nucleus with DAPI (Sigma‐Aldrich). Samples were fixed with 1% formaldehyde (Merck) in PBS (Lonza), and cover slides were attached with mounting medium. Samples were analysed using confocal laser scanning microscopy (SP5, Leica).

### 4.8 Stimulation of moDCs with gamma‐irradiated bacteria

*S*. *aureus* strains at exponential growth phase were washed with PBS and resuspended in PBS with addition of glycerol. Gamma irradiation of bacteria was performed by Synergy Health Ede B.V., a STERIS company (Ede, The Netherlands), and loss of viability was verified by culture. Concentrations of all bacterial suspensions were measured using MACSQuant Analyzer 10.

Immature moDCs were harvested, washed, and resuspended in RPMI +5% FBS. Before use, cells were stained for expression of MGL and maturation markers using MGL‐PE, CD80‐PE, CD83‐APC, CD86‐APC (all SONY Biotechnology), CD40‐FITC, and HLA‐DR‐APC (both BD Biosciences) antibodies and their corresponding isotype controls (BD Biosciences), diluted according to the manufacturers' instructions. Samples were analysed using flow cytometry. Bacteria were diluted in RPMI +5% FBS and mixed with 0.5 × 10^5^ immature moDCs in 1:2, 1:5, and 1:10 cell‐to‐bacteria ratios. Suspensions were incubated in Corning 96‐well round bottom ultra‐low attachment plates (Sigma‐Aldrich) for 16 hr at 37°C with 5% CO_2_. For blocking, moDCs were incubated for 16 hr with *S*. *aureus* PS187 WT at 1:2, 1:5, and 1:10 cell‐to‐bacteria ratios in the presence of anti‐MGL blocking antibodies (ASGPR/MGL, clone 125A10.03, Dendritics) or isotype control antibodies (produced and purified in‐house) at the concentration 10 μg/ml. Supernatants were collected after centrifugation, and cells from 1:10 cell‐to‐bacteria conditions were stained as described previously. IL‐8, IL‐12, and TNFα concentrations of the collected supernatants were analysed by Luminex assay.

### 4.9 Statistical analysis

Data obtained from flow cytometry was analysed using FlowJo 10 (FlowJo LLC). Statistical analysis of data was performed using GraphPad Prism 7.02 (GraphPad Software). One‐way analysis of variance followed by Dunnett's or Tukey's test or two‐way analysis of variance followed by Tukey's test were performed. Only significant differences between samples (*p* < .05) were indicated on graphs.

## AUTHOR CONTRIBUTIONS

M. E. M., R. v. D., A. P., and N. M. v. S. planned the experiments. M. E. M., A.H. and D. G. performed the experiments and analysed the data. M. E. M. performed statistical analysis. G. X. analysed *Staphylococcus lugdunensis* genome, D. G. did molecular cloning, SaPI transfer assay and WTA isolation, and PAGE analysis, and A. P. provided the bacterial strains. M. E. M. and N. M. v. S. wrote the manuscript. All authors revised and approved the manuscript.

## CONFLICT OF INTERESTS

M. E. M is a PhD fellow and is enrolled in the Infection and Immunity PhD programme, part of the Graduate school of Life Sciences at the University of Utrecht and participated in a postgraduate studentship programme at Glaxo Smith Kline (GSK).

## Supporting information


**Figure S1.** Binding of FITC‐labeled soy bean agglutinin (SBA) to S. aureus PS187 WT, GN1 mutant and coagulase negative staphylococci. Bars represent mean of fluorescence intensity ± SEM from three independent experiments.

**Figure S2.** Relative expression of surface maturation markers CD40 and HLA‐DR on human moDCs 16 h after stimulation with gamma‐irradiated S. aureus strains in 1:10 cell‐to‐bacteria ratio. Data are presented as fold change in fluorescence intensity ± SEM relative to unstimulated control.

**Figure S3.** Production of IL‐6, IL‐12p70 and TNFα by human moDCs 16 h after stimulation with gamma‐irradiated S. aureus PS187 WT in the absence or presence of anti‐MGL blocking antibody (αMGL) or isotype control antibody. Data are presented as fold increase over 1:2 cell‐to‐bacteria ratio for each cytokine. Mean ± SEM from three independent experiments using five different donors are shown.

Figure S4. Production of IL‐4, IL‐6, IL‐10, IL‐12p70, IL‐23p19 and TNFα by human moDCs after 16 h incubation in the absence or presence of anti‐MGL blocking antibody (αMGL) or isotype control antibody. None of the cytokines is significantly affected by presence of the antibodies.

**Table S1** Bacterial strains used in this study
