# Supplementary figures and images for: The C‐type lectin receptor MGL senses N‐acetylgalactosamine on the unique Staphylococcus aureus ST395 wall teichoic acid
Source: Cell Microbiol. 2019 Jul 8;21(10):e13072. doi: 10.1111/cmi.13072 (PMC6771913; doi:10.1111/cmi.13072)

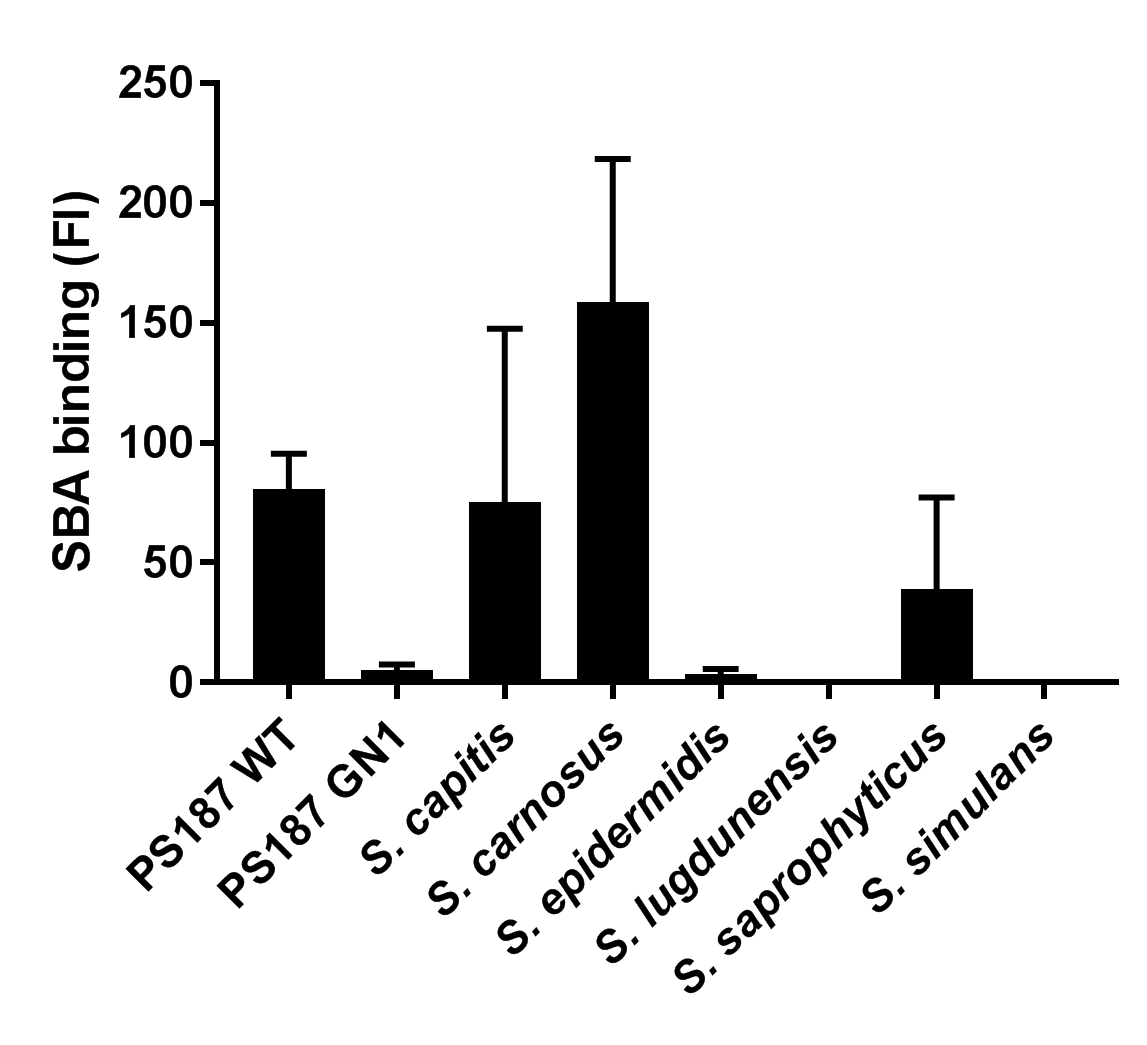

Supplement: Supplementary file 1 — Figure S1. Binding of FITC‐labeled soy bean agglutinin (SBA) to S. aureus PS187 WT, GN1 mutant and coagulase negative staphylococci. Bars represent mean of fluorescence intensity ± SEM from three independent experiments. [file CMI-21-na-s001.tif]

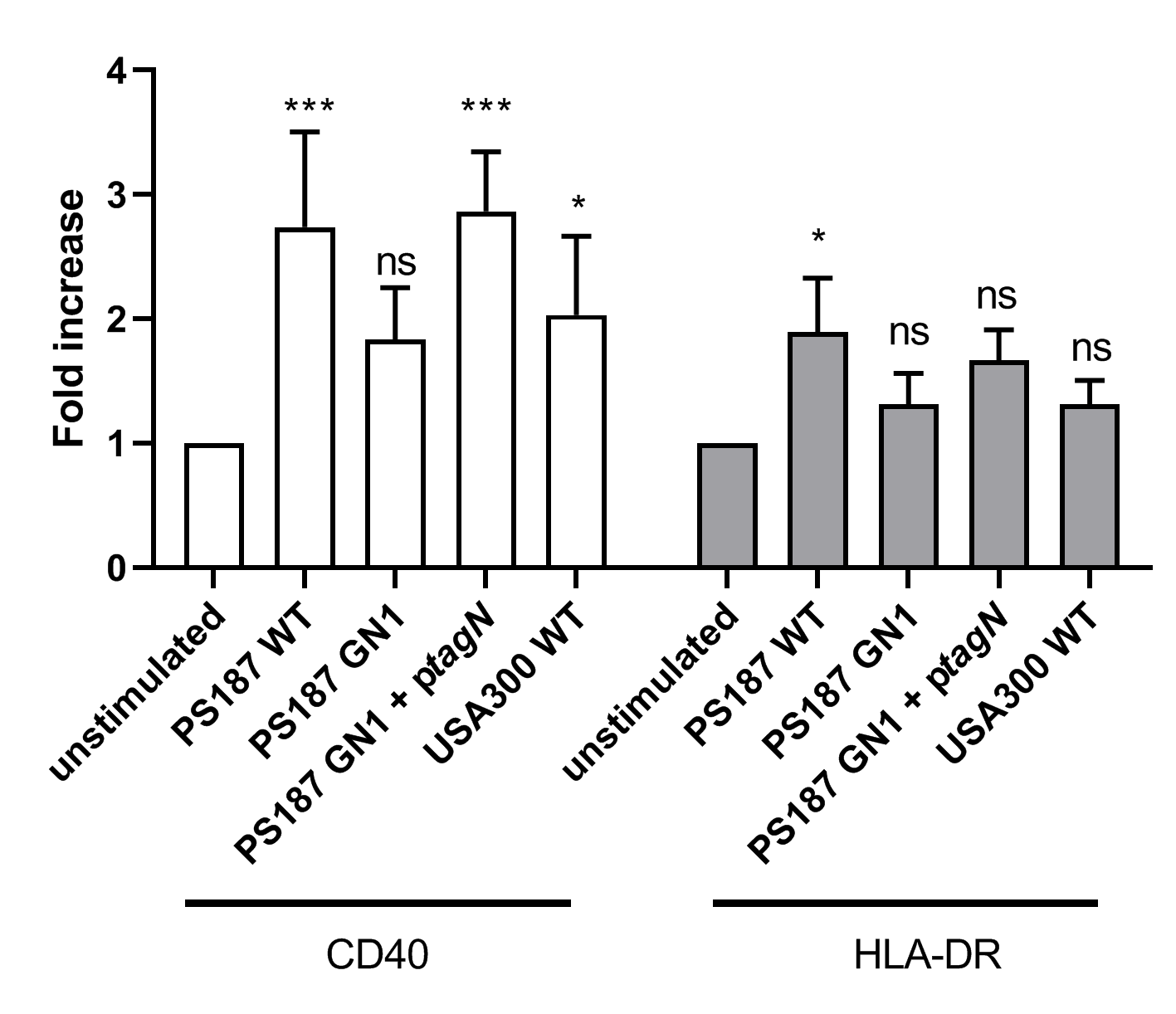

Supplement: Supplementary file 2 — Figure S2. Relative expression of surface maturation markers CD40 and HLA‐DR on human moDCs 16 h after stimulation with gamma‐irradiated S. aureus strains in 1:10 cell‐to‐bacteria ratio. Data are presented as fold change in fluorescence intensity ± SEM relative to unstimulated control. [file CMI-21-na-s002.tif]

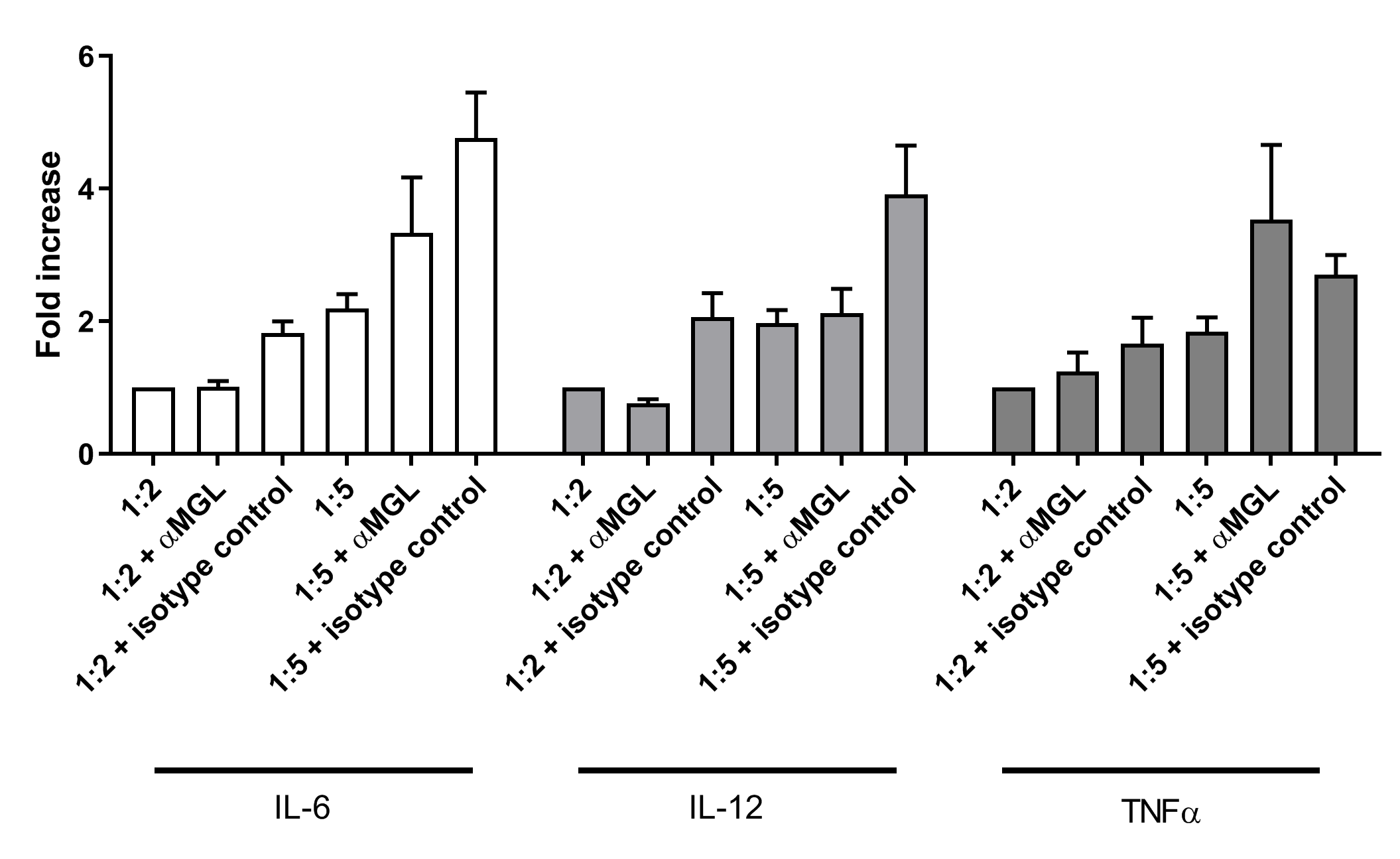

Supplement: Supplementary file 3 — Figure S3. Production of IL‐6, IL‐12p70 and TNFα by human moDCs 16 h after stimulation with gamma‐irradiated S. aureus PS187 WT in the absence or presence of anti‐MGL blocking antibody (αMGL) or isotype control antibody. Data are presented as fold increase over 1:2 cell‐to‐bacteria ratio for each cytokine. Mean ± SEM from three independent experiments using five different donors are shown. [file CMI-21-na-s003.tif]

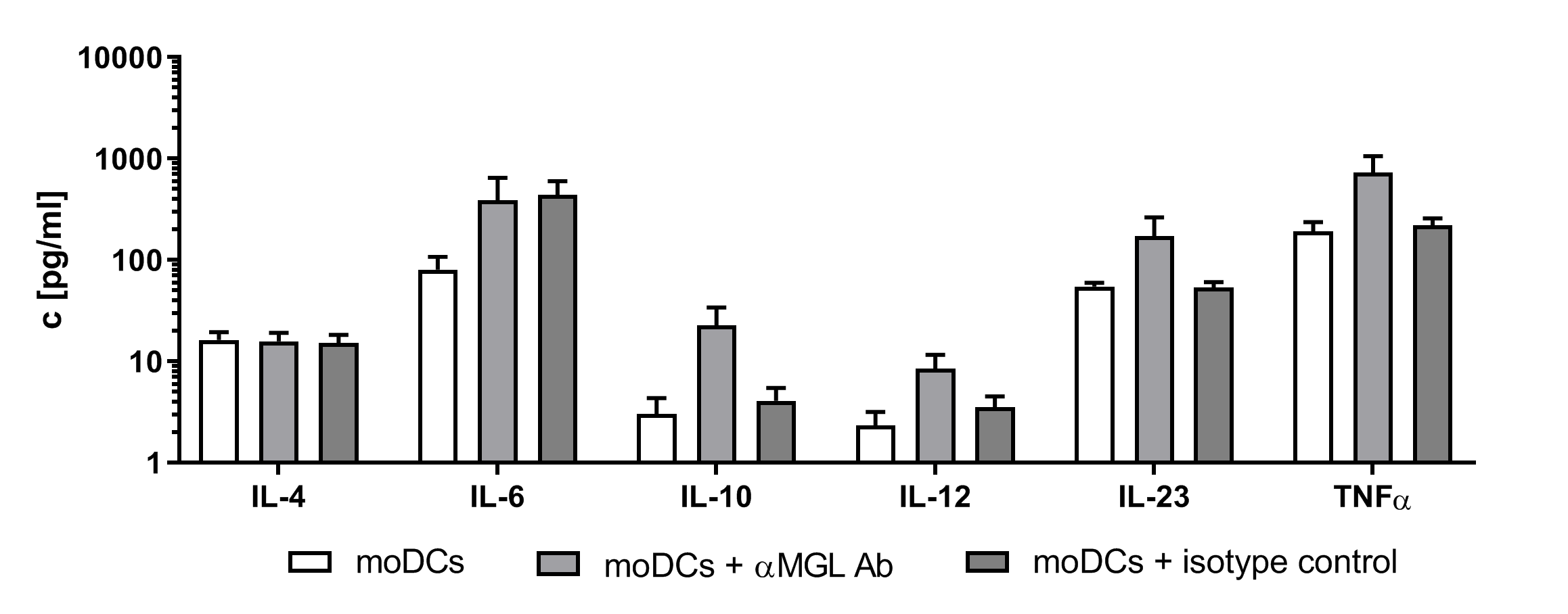

Supplement: Supplementary file 4 — Figure S4. Production of IL‐4, IL‐6, IL‐10, IL‐12p70, IL‐23p19 and TNFα by human moDCs after 16 h incubation in the absence or presence of anti‐MGL blocking antibody (αMGL) or isotype control antibody. None of the cytokines is significantly affected by presence of the antibodies. [file CMI-21-na-s004.tif]
