# Supplementary material for: The C‐type lectin receptor MGL senses N‐acetylgalactosamine on the unique Staphylococcus aureus ST395 wall teichoic acid
Source: Cell Microbiol. 2019 Jul 8;21(10):e13072. doi: 10.1111/cmi.13072 (PMC6771913; doi:10.1111/cmi.13072)
Supplement: Supplementary file 5 — Table S1 Bacterial strains used in this study [file CMI-21-na-s005.docx]

**Supplementary Figure legends**

Supplementary Figure S1. Binding of FITC-labeled soy bean agglutinin (SBA) to *S. aureus* PS187 WT, GN1 mutant and coagulase negative staphylococci. Bars represent mean of fluorescence intensity ± SEM from three independent experiments.

Supplementary Figure S2. Relative expression of surface maturation markers CD40 and HLA-DR on human moDCs 16 h after stimulation with gamma-irradiated *S. aureus* strains in 1:10 cell-to-bacteria ratio. Data are presented as fold change in fluorescence intensity ± SEM relative to unstimulated control.

Supplementary Figure S3. Production of IL-6, IL-12p70 and TNFα by human moDCs 16 h after stimulation with gamma-irradiated *S. aureus* PS187 WT in the absence or presence of anti-MGL blocking antibody (αMGL) or isotype control antibody. Data are presented as fold increase over 1:2 cell-to-bacteria ratio for each cytokine. Mean ± SEM from three independent experiments using three different donors are shown.

Supplementary Figure S4. Production of IL-4, IL-6, IL-10, IL-12p70, IL-23p19 and TNFα by human moDCs after 16 h incubation in the absence or presence of anti-MGL blocking antibody (αMGL) or isotype control antibody. None of the cytokines is significantly affected by presence of the antibodies.

Supplementary Table 1 Bacterial strains used in this study

| Strain | Source |
| --- | --- |
| *S. aureus* Newman wild type (ST254, CC8) | ATCC, Cat#13420 |
| *S. aureus* USA300 wild type (NRS384, ST8, CC8) | NARSA strain collection |
| *S. aureus* PS187 wild type (ST395, CC395) | ATCC, Cat#15564 |
| *S. aureus* PS187 GN1 | (Winstel *et al.*, 2014) |
| *S. aureus* PS187 GN1 + pRB *tagN* | (Winstel *et al.*, 2014) |
| *S. aureus* PS187 GN1 + pRB *S. lugdunensis tagN* | This study |
| *S. aureus* JS395 wild type (ST395, CC395) | (Francois *et al.*, 2007) |
| *S. aureus* T132-1 wild type (ST395, CC395) | (Holtfreter *et al.*, 2007) |
| *S. aureus* s1330002 wild type (ST395, CC395) | (Holtfreter *et al.*, 2007) |
| *S. aureus* T166-1 wild type (ST395, CC395) | (Holtfreter *et al.*, 2007) |
| *S. aureus* T110-1 wild type (ST395, CC395) | (Holtfreter *et al.*, 2007) |
| *S. aureus* T191-1 wild type (ST395, CC395) | (Holtfreter *et al.*, 2007) |
| *S. capitis* ATCC27840 wild type | ATCC, Cat#27840 |
| *S. carnosus* TM300 wild type | (Rosenstein *et al.*, 2009) |
| *S. epidermidis* 1457 wild type | (Mack *et al.*, 1992) |
| *S. lugdunensis* SL13 wild type | (Chassain *et al.*, 2012) |
| *S. lugdunensis* HKU09-01 | (Tse *et al.*, 2010) |
| *S. saprophyticus* ATCC 35552 wild type | ATCC, Cat#35552 |
| *S. simulans* ATCC 27848 wild type | ATCC, Cat#27848 |
| *S. aureus* PS187 SaPIbovI (tst::tet) | (Winstel *et al.*, 2014) |

**Supplementary references**

Chassain, B., Lemee, L., Didi, J., Thiberge, J.M., Brisse, S., Pons, J.L. and Pestel-Caron, M. (2012). Multilocus sequence typing analysis of Staphylococcus lugdunensis implies a clonal population structure. *J Clin Microbiol* **50,** 3003-3009.

Francois, P., Bento, M., Renzi, G., Harbarth, S., Pittet, D. and Schrenzel, J. (2007). Evaluation of three molecular assays for rapid identification of methicillin-resistant Staphylococcus aureus. *J Clin Microbiol* **45,** 2011-2013.

Holtfreter, S., Grumann, D., Schmudde, M., Nguyen, H.T., Eichler, P., Strommenger, B.*, et al.* (2007). Clonal distribution of superantigen genes in clinical Staphylococcus aureus isolates. *J Clin Microbiol* **45,** 2669-2680.

Mack, D., Siemssen, N. and Laufs, R. (1992). Parallel induction by glucose of adherence and a polysaccharide antigen specific for plastic-adherent Staphylococcus epidermidis: evidence for functional relation to intercellular adhesion. *Infect Immun* **60,** 2048-2057.

Rosenstein, R., Nerz, C., Biswas, L., Resch, A., Raddatz, G., Schuster, S.C. and Gotz, F. (2009). Genome analysis of the meat starter culture bacterium Staphylococcus carnosus TM300. *Appl Environ Microbiol* **75,** 811-822.

Tse, H., Tsoi, H.W., Leung, S.P., Lau, S.K., Woo, P.C. and Yuen, K.Y. (2010). Complete genome sequence of Staphylococcus lugdunensis strain HKU09-01. *J Bacteriol* **192,** 1471-1472.

Winstel, V., Sanchez-Carballo, P., Holst, O., Xia, G. and Peschel, A. (2014). Biosynthesis of the unique wall teichoic acid of Staphylococcus aureus lineage ST395. *mBio* **5,** e00869.
